# Integrated Analysis of microRNA Targets Reveals New Insights into Transcriptional–Post-Transcriptional Regulatory Cross-Talk

**DOI:** 10.3390/biology14010043

**Published:** 2025-01-08

**Authors:** Simona Panni, Roberto Pizzolotto

**Affiliations:** Dipartimento di Biologia Ecologia Scienze della Terra (DiBEST), Università della Calabria, 87036 Rende, CS, Italy; roberto.pizzolotto@unical.it

**Keywords:** microRNA, biocuration, miRNA interaction network, gene regulation, transcription factors

## Abstract

It is becoming increasingly clear that microRNAs are key players in gene regulatory networks, modulating gene expression at post-transcriptional level. Their involvement in almost all cellular processes predicts their role in diseases, and several microRNA-based therapeutics are currently undergoing clinical testing. Despite their undeniable relevance and the substantial body of literature demonstrating their role in cancer and other pathologies, the identification of functional interactions is still challenging. To address this issue, several resources have been developed to collect information from the literature, according to different criteria and reliability scores. In the present study, we have constructed a network of verified microRNA-mRNA interactions by integrating strong-evidence couples from different resources. Our analysis of the resulting network reveals that only one-fifth of the human genes exhibits experimental validated regulation by microRNAs. A very small subset of them is controlled by more than 20 microRNAs, and these hubs are highly enriched of pivotal transcription factors and regulatory proteins, strongly suggesting a complex interplay and a combinatorial effect between transcriptional and post-transcriptional gene control. Data analysis also reveals that several microRNAs control multiple targets involved in the same pathway or biological process, likely contributing to the coordinated control of the protein levels.

## Simple Summary

MicroRNAs are short RNA molecules performing crucial regulatory roles in human cells, making them an attractive target for synthetic drugs that can either replace or inhibit them. However, perturbing the microRNA network by enhancing or depleting a single miRNA often leads to unpredictable outcomes. This is partly due to our incomplete understanding of the full set of genes regulated by each miRNA. To address this challenge, we have proposed a set of criteria to retrieve from public repositories the most reliable interactions, and we have used the resulting network to gain more insights into general microRNA regulatory mechanisms. We show that microRNAs exert a massive control on key regulatory genes, with more than 20 microRNAs acting on the same gene and possibly coordinating different genes in the same pathway.

## 1. Introduction

Over the past two decades, genome projects have shown that the complexity of the most evolved organisms cannot be fully explained looking at protein-coding genes alone, as they are highly conserved even in distantly related species. Large genomic regions are occupied by genes for the so-called non-coding RNAs, which perform their functions without being translated into amino acid chains, and that may contribute significantly to the variability among different organisms. Among them, microRNAs have emerged as key regulators since their first discovery in 1993, and as an essential component of the intricate network that regulates gene expression in many cellular processes [1,2]. Their mature form is a very short double stranded RNA, composed of a passenger strand that is displaced, and a guide strand that identifies the target, commonly through base pairing between nucleotides in positions 2–7 of the microRNA (the seed) and a short complementary region in the mRNA, often located in 3′ UTR of the transcript [3,4]. The peculiar shape of microRNAs has promoted the development of many computational approaches, both to discover them in the genome and to predict their targets. Currently, more than a hundred target prediction tools are available, relying on sequence complementarity and other features, as extensively reviewed in the literature [5,6,7]. According to bioinformatics analyses, almost all mRNAs contain several sequences that are potentially recognised by microRNAs; however, only a small part of them have been experimentally validated [5]. The binding between each microRNA and the selected mRNA occurs within the miRNA-induced silencing complex (miRISC) and leads to decreased protein expression through translational repression and/or mRNA degradation. Briefly, when the microRNA is loaded onto an Argonaute protein, the passenger strand is released and the guide strand hybridises to the complementary region on the mRNA [3]. The TNR6A protein (also known as GW182), binds to the Argonaute complex, facilitating the recruitment of the CCR4-NOT and PAN2-PAN3 deadenylase complexes as well as decapping enzymes [8,9]. While several studies report microRNA-mediated translational repression, mRNA decay is considered the primary regulatory mechanism [8]. This is supported by the observation that the majority of the studies demonstrating microRNA-mRNA interaction by luciferase assay [10,11] also observe a concomitant decrease in the mRNA level upon miRNA mimic transfection. Several RNA binding proteins contribute to the outcome of the regulatory process by recognising specific sequences or secondary structures on the 3′ UTR of the mRNAs [12]. These proteins can either mask the seed sequence or facilitate the microRNA binding, partially explaining the discrepancy between the set of predicted versus validated targets.

A growing body of evidence points to the key role of microRNAs in gene regulation, and some of them control multiple genes that are altered in pathological conditions; therefore, they are under investigation to develop new therapeutic strategies [13]. Accurate knowledge of the web of interactions is crucial to foresee the downstream effects of microRNA-based therapies. To this aim, several repositories collect microRNA interaction data, and considerable efforts have been made to standardise annotation vocabularies and RNA sequences identifiers to facilitate data integration [14,15,16]. Nonetheless, significant discrepancies persist among different databases, regarding which experimental approaches demonstrate “true” interactions, especially for high-throughput assays. Immunoprecipitation of AGO2 in Clip-seq experiments generates lists of co-immunoprecipitated microRNAs and mRNAs, and some resources capture these results as interaction pairs, while others recognise their value in improving the performance of prediction tools but not as proof of interaction [17]. An adaptation of this technique, involving the ligation of RNA duplexes before sequencing, was proposed, to specifically investigate on microRNA targets [18,19].

In recent years, hundreds of papers have been published showing the physical binding of a microRNA to its partner by luciferase assay, performed on the wild type and on the mutated mRNA. This approach, along with RNA immunoprecipitation (which is less frequently employed) is widely accepted as evidence of binding between the two RNAs [15,20]. The UCL functional annotation group [14,21] and the IntAct database [22] have annotated a collection of such interactions, although the coverage is still limited. MirTarBase [23] and RNAInter [24] offer broader coverage, enabling the selection of the most robust evidence from the other annotations. To investigate the key features of the microRNA mediated regulation, we constructed a network based on the most reliable interactions and analysed the main characteristics of the resulting post-transcriptional regulatory circuit.

## 2. Materials and Methods

### 2.1. Dataset Integration

**Figure 1:**
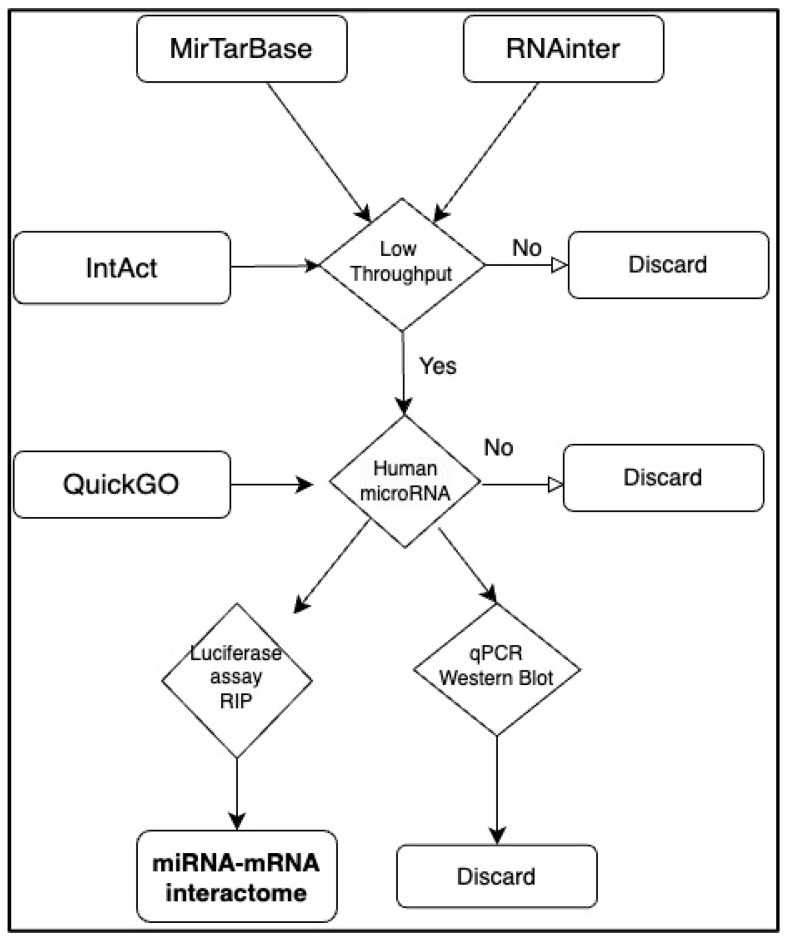
Quality control pipeline for microRNA-mRNA interaction network. Data were downloaded from IntAct, miRTarBase, QuickGO and RNAinter and filtered for low-throughput experiments which demonstrate the direct binding.

Human microRNA-mRNA interactions were downloaded from four repositories in Jan 2024: miRTarBase [25] RNAinter [24] QuickGO [14,26] IntAct [22]. Interactions from QuickGO and IntAct are consistent to the International Molecular Exchange consortium common standards (https://www.imexconsortium.org/ (accessed on 1 December 2024)) [27], while interactions derived from miRTarbase and RNAinter were filtered for low-throughput, strong evidence. More precisely, to assure the physical binding of the microRNA to the mRNA, we selected “luciferase assays” or RNA-IP and disregarded “western blot” and “qpcr” as these can be the result of a direct or indirect effect (Figure 1 [15]) To integrate the data, the Uniprot ID mapping tool (https://www.uniprot.org/id-mapping (accessed on 30 January 2024)) [28] was used when necessary. Each interaction was considered once and then all duplications were deleted. The Cytoscape tool [29] was used to visualise and analyse the networks. The list of DNA-binding transcription factors was retrieved from [30]. To obtain an estimation of the number of genes regulated by each transcription factor we downloaded gene regulation data (TF-TG) from the TFlink “small scale dataset” filtering out inferred interactions [31]. This should contain only interactions verified in low-throughput experiments. TF-regulating zero genes according to this list were deleted.

The microRNA-mRNA network was constructed and the number of edges for each node (defined as node degree) was calculated [29]. Degree classes (dc) were calculated based on how many times a certain degree appears (abundance). As an example, if there are two nodes with 94 interactions each one, then degree 94 belongs to the degree class 2. Three datasets were produced, listing the abundance of dc relative to mRNA, miRNA and for the subclass of transcription factors (see below). To compare the abundance distribution of the datasets, the regression model best fitting each abundance distribution was computed on the basis of several models proposed in the R library “sads” [32]. The best model was selected based on AIC, and whether the abundance distributions converged on the same model, this was taken as evidence of similarity. We compared the abundance distribution of the transcription factors (TFs) with that of all mRNAs. Transcription factors regulated by zero microRNAs were deleted and the others were subtracted from the mRNA dataset, and the two curves were compared by means of the AIC value.

Mutual Information (MI) analysis was applied for the effective estimation of the possible relationship between the number of microRNAs regulating each TF and the number of genes regulated by the same TF, by means of the R library “entropy” [33]. MI can be applied to assess the presence of an unspecified relationship among datasets, when a linear model is not applicable. It measures the amount of information obtained about one variable when measuring the other [34,35]. MI returns an absolute value, which can be more easily interpreted if expressed as relative MI (rMI), i.e., the ratio between MI and the maximum MI that can be extracted for the dataset [36]. To evaluate confidence intervals (CI) and significance (p), MI and rMI were bootstrapped by means of the R libraries “simpleboot”, “boot” and “boot.pval”.

### 2.2. Gene Ontology and Pathway Enrichment Analysis

Genes interacting with more than 20 microRNAs were searched for Gene Ontology terms enrichment, in comparison to the whole genome. To this aim, Gprofiler [37], VLAD [38] (moved to https://www.informatics.jax.org/vlad/ (accessed on 1 February 2024)) and BinGO [39] were utilised, giving very similar results. The data presented show the enrichment for molecular function obtained with BinGO, when the significance was imposed below 0.0005 and the whole genome was considered benchmarking. Supplementary Figure S1 was drawn with VLAD. Pathway enrichment analyses were conducted with Reactome analysis tool [40]

### 2.3. Other Statistical Analysis

Intersections of common target genes were calculated with https://bioinformatics.psb.ugent.be/webtools/Venn/ (accessed on 1 July 2024).

To correlate 3′ UTR lengths with predicted and verified interactions, data tables were generated as follows: transcript lengths and 3′ UTR lengths were extracted from the Ensembl (https://www.ensembl.org/index.html (accessed on 1 March 2024)) and refer to the “main” transcript as defined by the GIFT tool (https://www.ebi.ac.uk/gifts/ (accessed on 1 March 2024)). The number of interactions was extracted from the integrated dataset described above. Predictions were calculated with miRDB [41] and miRWalk [42]. miRwalk prediction was calculated on the same Refseq as for the UTR and length measures and filtered at 0.95. Pearson correlation coefficient was calculated using the ggpairs functions from GGally package [43] in R [44].

## 3. Results

### 3.1. General Characteristics of the microRNA-mRNA Network

Although any gene is a potential target for microRNAs, as suggested by numerous target prediction methods, low-throughput experiments seem to reveal a significant discrepancy among transcripts. In a recent paper, the collection from the literature of microRNAs which regulate genes involved in rare diseases has shown that for the majority of mRNAs no microRNA binding has been demonstrated, while a small number of mRNAs are heavily regulated [15]. To gain more insights into the distribution of microRNA targets, we have integrated four manually curated datasets [14,22,24,25] and filtered them to include only strong-evidence, low-throughput results, as described in detail in Figure 1. We used the resulting list of “gold standard” interactions to investigate post-transcriptional regulatory mechanisms.

**Figure 2:**
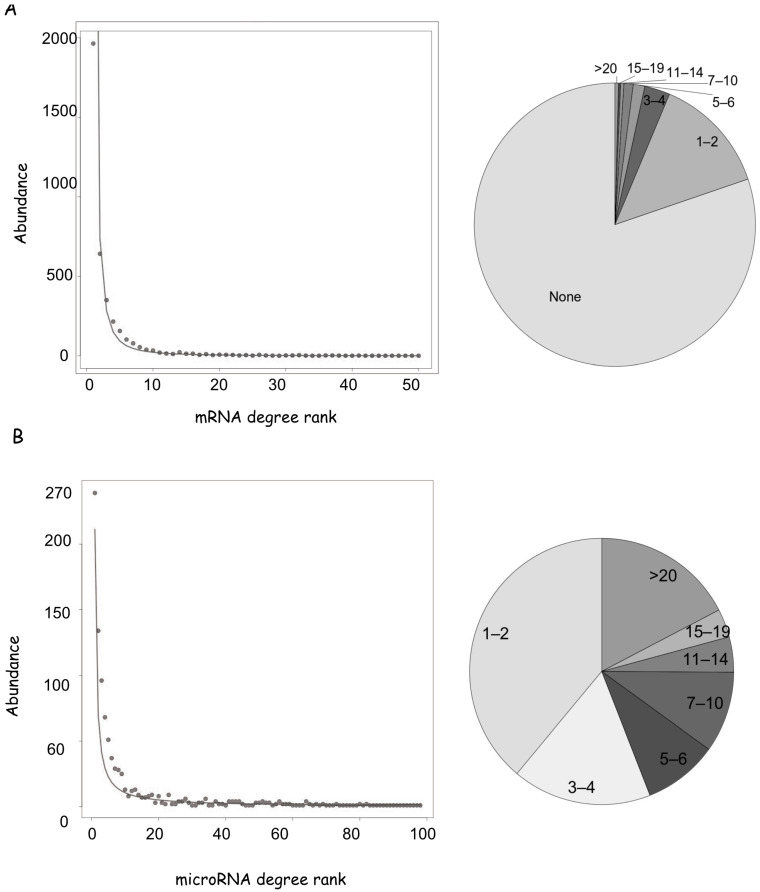
Properties of the human mRNA-microRNA network. (**A**) mRNA degree distribution fitting a power law. Degrees were divided in classes corresponding to interactor numbers and the rank of each class was plotted against its abundance. On the right is a pie chart of the mRNA network, showing the frequencies of degree classes in different grey scales. For convenience classes, they were grouped as indicated. (**B**) microRNA degree distribution and pie-chart of the microRNA network, as in (**A**).

According to this dataset, 3842 human genes are regulated by at least one microRNA, which corresponds to 20% of the estimated 19,400 human coding genes (www.genenames.org (accessed on 1 March 2024)). We calculated the number of edges for each node (defined as node degree, dc). Concerning the mRNA nodes, the smallest class, with dc = 1, contains 1964 presences, i.e., more than 1900 mRNAs interact with one microRNA; while the largest class with dc = 108 is present once (and corresponds to PTEN phosphatase). The abundance decreases quickly between 5 and 10 degrees so that a minority of nodes have more than 10 interactors (Figure 2A, Supplementary Table S1). This scenario is consistent with the abundance distribution best fitting a power law, where large values are exponentially less likely, and a large proportion of occurrences is held by a small number of entities (Pareto distribution). Only 2% of the 3842 mRNAs regulated by microRNAs are controlled by more than 20 microRNAs, and we defined them as network hubs for the following analyses (Supplementary Table S1).

The miRNA dataset lists 962 microRNA nodes, which have an average higher degree in comparison to mRNAs. The smallest class with dc = 1 shows 239 presences and 14% of interacting microRNAs regulate more than 20 genes. Notably, thirteen microRNAs target more than 100 genes each (Figure 2B, Supplementary Table S1), and this number is destined to increase as new data will be published.

In synthesis, the mRNA expression level of a small number of genes is under control of multiple microRNAs to finely modulate them in different tissues, while many transcripts may not be affected by microRNA regulation. Most of the microRNAs with validated targets control more than two genes and a significative portion binds to multiple genes, possibly coordinating their expression.

### 3.2. mRNA Highly Regulated Are Regulators

**Figure 3:**
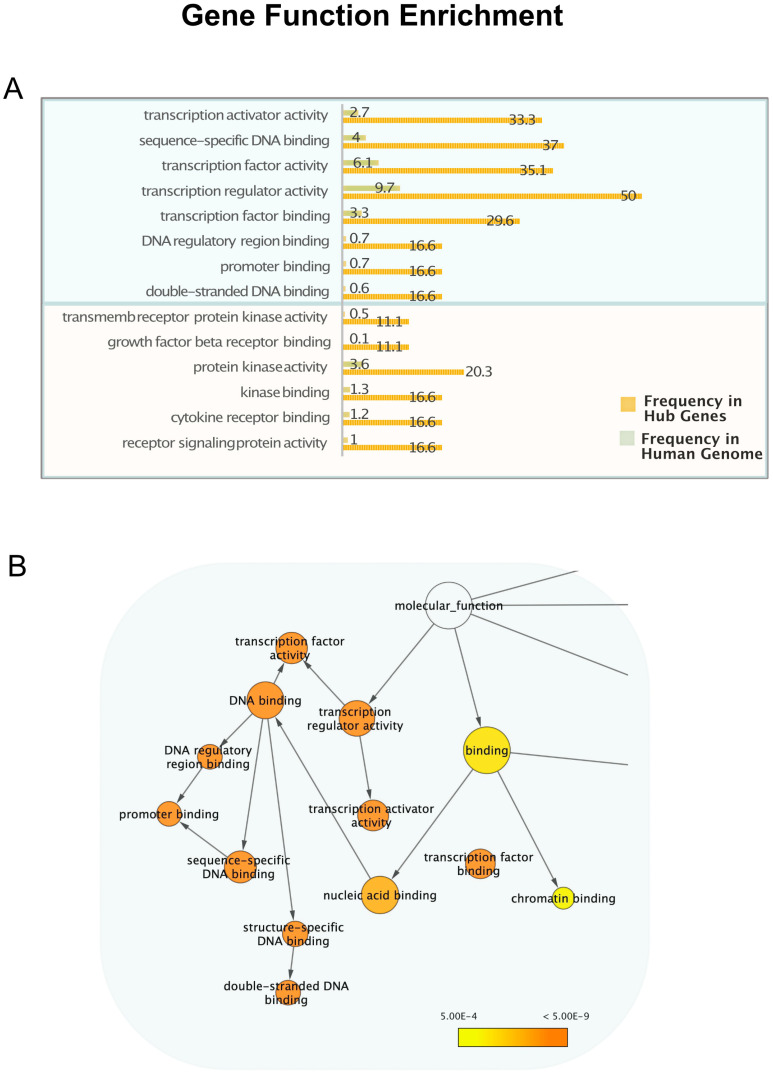
Transcription regulation function in genes controlled by more than 20 microRNAs. (**A**) The histogram presents the molecular function GO terms statistically overrepresented (significance level 0.0005) in the hub genes compared to the whole genome, clearly showing an enrichment for genes related to transcription regulation and signal transduction. The complete list of molecular functions enriched in hub genes and their frequencies are listed in Supplementary Table S2. (**B**) Ontology graph drawn with BinGO in Cytoscape [33], showing the GO hierarchy of enriched child terms related to transcription. The area of a node is proportional to the number of genes annotated to the corresponding GO category among the “hubs” and the colours indicate the statistical significance, higher in dark orange nodes.

To gain more insights into microRNA-mediated post-transcriptional regulation, we investigated the molecular functions of the highly regulated genes to determine whether specific gene types have a higher propensity to be controlled by multiple miRNAs. Gene Ontology is the most used bioinformatic resource to unify and interpret gene functions and attributes for biomedical purposes [21]. We calculated the enrichment in genes performing specific functions among those regulated by more than 20 microRNAs, in respect to the whole set of human genes, using three different tools: Gprofiler [37], VLAD [38] and BinGO [39]. These three methods gave similar results, showing a very high enrichment for genes related to transcription regulatory activity. Figure 3 shows Ontology Terms related to the transcription and transcription regulation, highly enriched among “hub” genes in respect to the others. In Supplementary Table S2, all the enriched terms retrieved with BinGO are listed with their frequency in hub genes and in the whole genome. The Reactome analysis tool [40] also returns among the top-ranking pathways, “Generic Transcription Pathway” and “RNA Polymerase II Transcription” and “Gene Expression”, all with FDR 1.05 × 10^−14^.

Notably, a similar result had been obtained in [45] from the analysis of hub genes predicted with Targetscan [46].

**Figure 4:**
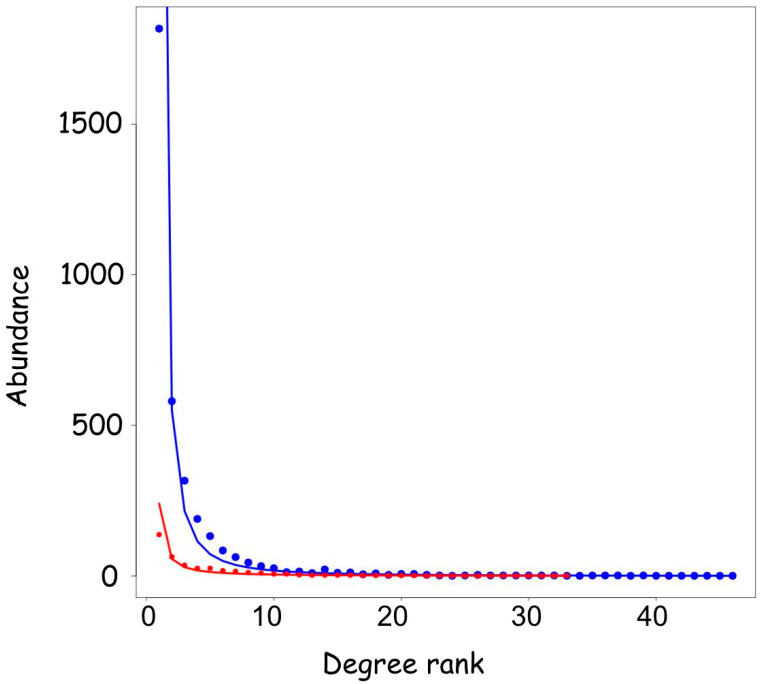
The number of microRNAs interacting with transcription factors versus all the other genes. The degree distribution of the mRNA dataset (except transcription factors) (blue line) is compared with the transcription factor dataset (red line). The two datasets are represented by the same model, and the TF dataset has an AIC value of 174 indicating that it is even better represented by the power law curve in respect to the mRNA dataset with AIC = 401.

On the other hand, not all the transcription factors are regulated by microRNAs. In a recent paper, members of the GO Consortium have produced a curated catalogue of 1456 human DNA-binding transcription factors [30]. We compared the distribution of the interacting microRNAs of these 1456 genes with the whole set of human mRNAs, and we found a very similar distribution (Figure 4). For 72% of the DNA-binding TFs listed in [30], no interacting microRNA were reported, while 28% have at least one. Only 25 transcription factors were regulated by more than 20 microRNA, corresponding to 1.7%. The rank abundance distribution of microRNAs binding to transcription factors fits a Pareto distribution for the other mRNAs, with a minimum value of AIC = 174 as compared to AIC= 401 for the other genes, indicating that they are represented by a power law distribution.

To better estimate if there is any relationship between the microRNA regulation and the transcriptional regulation, we performed a Mutual Information analysis, considering, for each transcription factor, two variables: the number of interacting microRNAs and the number of genes regulated by. To estimate the number of genes regulated by each transcription factor we referred to TFlink [31]. The MI value (MI = 0.1161408) and the rMI value (rMI = 0.02567784) both fall in a range within the CIs with a highly significant *p*-value, indicating a non-random co-occurrence. However, the low rMI value indicates that other variables influence the numbers.

From these observations we conclude that, although genes controlled by multiple microRNAs are highly enriched for transcription regulation functions, not all the transcription factors are regulated by microRNAs. As shown in Supplementary Table S3, however, the most interconnected factors are highly controlled by microRNAs.

**Figure 5:**
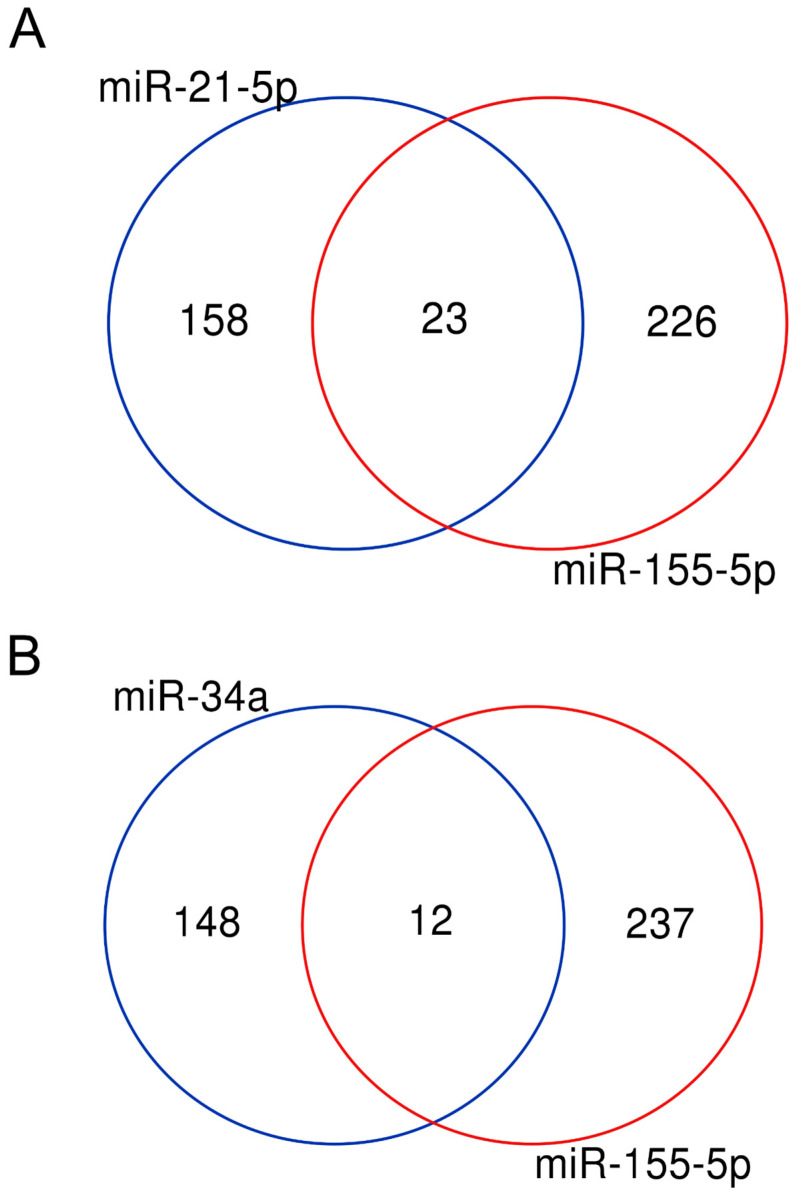
OncomiR and suppressor microRNAs share common targets. (**A**) Venn diagram showing the overlapping genes regulated by two oncomiRs: miR-155 and miR-21. The 23 common targets are dock1 pten myd88 nef pik3r1 apaf1 egfr msh6 socs6 smarca4 bcl2 bcl6 thrb cebpb bcl10 icam1 jun foxo1 socs1 vhl msh2 casp3 sox6. (**B**) Venn diagram showing the overlapping genes regulated by the tumour suppressor miR-34a and the oncomiR miR-155. The 12 common targets are xiap, nfe2l2, myb, spi1, rad51, csf1r, bcl2, agtr1, cebpb, bcl6, myc, ccnd1.

Genes involved in receptor signalling and kinase binding are also enriched among microRNA-regulated genes (Figure 3A), and the proteins they code for are highly interconnected in PPI networks. For example, considering the term “protein kinase activity”, the genes annotated for this term code for the proteins gsk3b cdkn1a cdk6 ccnd1 erbb2 akt1 met tgfbr1 egfr igf1r tgfbr2, all interacting with hundreds of proteins, according to IntAct database [22]. Interestingly, the mRNA which interacts with the highest number of microRNAs, more than one hundred, encodes for the Phosphatase and Tensin Homolog PTEN, a well-known tumour suppressor gene, which inhibits the PI3K/AKT growth signalling. PTEN is downregulated by several “oncogenic” microRNAs (see Figure 5) which exert their tumorigenic function through this key phosphatase [47].

Supplementary Figure S1 shows the enrichment diagram obtained by VLAD [38], where *p* values for enriched terms are reported.

### 3.3. microRNAs Regulate Multiple Targets

It has been proposed that one of the main functions of microRNAs is to modulate gene expression to finely coordinate the levels of proteins involved in the same biological process or pathway [48,49]. According to the dataset presented in this work, 590 microRNAs control more than two targets and 13, listed in Table 1, regulate more than 100 transcripts each (Supplementary Table S1). Many microRNAs are associated with cancer or other pathologies and their expression is deregulated in the affected cells [50]. Table 1 shows that a miRNA controlling one hundred or more genes is a “oncomiR” or “tumour suppressor”, which means that it promotes or represses cancer phenotype, respectively. Despite their opposite effects, these microRNAs have some common targets: the Venn diagram in Figure 5 shows that 23 targets are in common between miR-155 and miR-21 (both described as oncomiR) and 12 genes are regulated by miR-155 (oncomiR) and miR-34a (tumour suppressor).

**Table 1:** Function of the most interconnected microRNAs in cancer.

microRNA Name	Number of Interactors	Oncogene	Tumour Suppressor	Reference
hsa-miR-155-5p	262	YES		[50]
hsa-miR-21-5p	182	YES		[51]
hsa-miR-145-5p	171		YES	[13]
hsa-miR-34a-5p	156		YES	[50]
hsa-miR-125b-5p	141		YES	[52]
hsa-miR-124-3p	138		YES	[52]
hsa-miR-29b-3p	135		YES	[52]
hsa-miR-200c-3p	134		YES	[13]
hsa-miR-17-5p	131	YES		[53]
hsa-miR-29a-3p	127		YES	[52]
hsa-miR-1-3p	110		YES	[52]
hsa-miR-20a-5p	107	YES		[53]
hsa-miR-9-5p	103		YES	[52]

Interestingly, miR-155-5p is the most connected microRNA in the network, and it regulates approximately 260 genes. It is considered an oncomiR as it was found overexpressed in lymphoma, leukaemia and several solid tumours. In normal tissues, it is expressed at a very low level with the exception of hematopoietic cells, lymph nodes and spleen, which suggests its implication in inflammatory response. Indeed, pathway analysis on its targets shows that the list of regulated genes is enriched in proteins involved in Interleukin-4 and Interleukin-13 signalling (15 genes), signalling by Interleukins (33 proteins), Cytokine Signalling in Immune system (39 proteins) and Immune System (68 proteins) (FDR 5.4 × 10^−11^, 3.51 × 10^−10^, 1.66 × 10^−7^ and 5.5 × 10^−4^, respectively). It is also significantly enriched in both TLR3 and TLR4 cascade with 11 and 13 proteins, respectively. The most enriched pathways in miR155-5p targets (and other microRNAs listed in Table 1) are listed in Supplementary Table S4.

**Figure 6:**
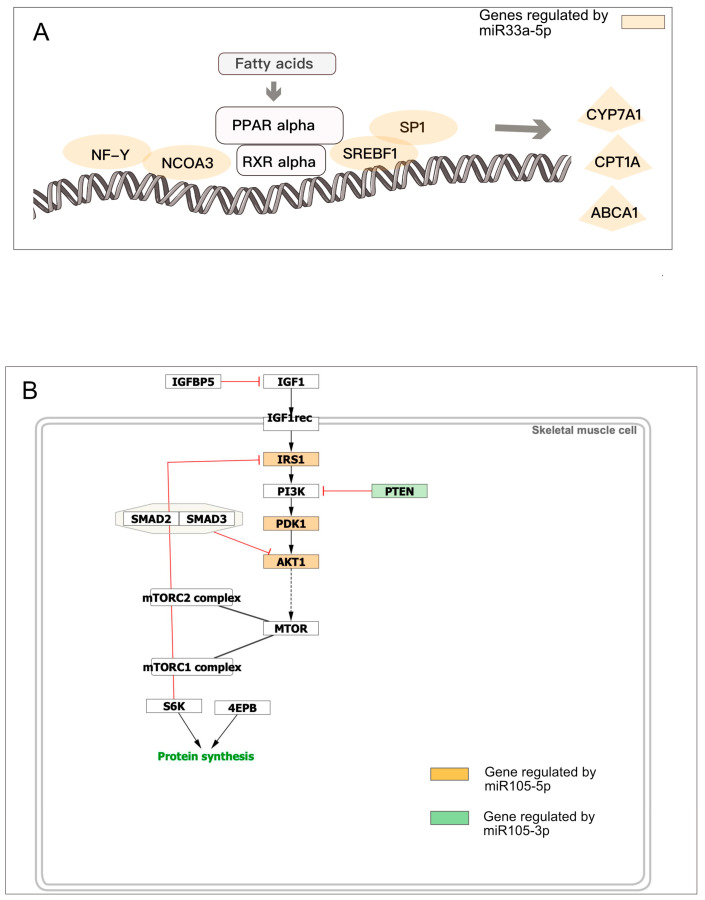
microRNAs regulate pathways by targeting multiple genes. (**A**) miR-33a regulates seven genes involved in the “Regulation of lipid metabolism by PPARalpha” R-HSA-40020 pathway; (**B**) miR105 targets four genes in “Factors and Pathways Affecting Insulin Like Growth Factor IGF1 Akt Signaling” pathway.

It has been suggested that genes regulated by the same microRNA may be involved in the same pathway to orchestrate the coregulation [54,55]. Consistently, we observed that several microRNAs controlling multiple genes target more entities of a specific pathway. For example, miR206 regulates 6 genes (out of 15) involved in the Reactome “NFE2L2 regulates pentose phosphate pathway” (R-HSA-9818028, FDR 6.18 × 10^−8^), or miR-33a regulates 7 genes involved in “Regulation of lipid metabolism by PPARalpha” (R-HSA-400206, FDR 5.3 × 10^−9^) as shown in Figure 6A. Interestingly, miR-105-5p downregulates three genes involved in the pathway, “Factors and Pathways Affecting Insulin Like Growth Factor IGF1 Akt Signaling”, while miR-105-3p, transcribed from the complementary strand, downregulates the hub PTEN, involved in the same pathway (Figure 6B).

### 3.4. The Length of the Untranslated Regions Do Not Correlate with the Abundance of Interactors

Most human protein-coding genes contain regions complementary to the seed sequences of the microRNAs that potentially mediate their binding. The seeds are short nucleotide sequences (approximately 6–7 nucleotides), so complementary stretches may occur by chance in the transcripts without reflecting any regulatory function. For example, six nucleotides may occur accidentally every 4096 nucleotides, and the longer the transcript sequence, the more likely it contains potential microRNAs matching sequences. The number of potential binding sites is even larger considering that the base pairing rarely needs a perfect match in animals, but few mismatches are tolerated [5,9]. Interestingly, the average length of the 3′ untranslated regions UTR have been correlated with species complexity, even within vertebrates [56,57]. Moreover, it has been demonstrated that different transcript isoforms, bearing longer/shorter UTRs, may be differentially regulated by microRNAs [58,59].

We performed a simple test, comparing the 3′ UTR length of a sample of transcripts with the number of interactors experimentally verified and the number of predicted binding sites to verify if more regulated mRNAs bear longer untranslated regions. The sample included highly connected mRNAs as well as transcripts with no verified interactors (Supplementary Table S5). To estimate the predicted binding sites, we selected two high-performance prediction tools, MiRDB [41] and MirWalk [42], both using additional criteria besides the matching sequence, spanning from deep learning from high-throughput data and expression profiling data to energetic considerations.

**Figure 7:**
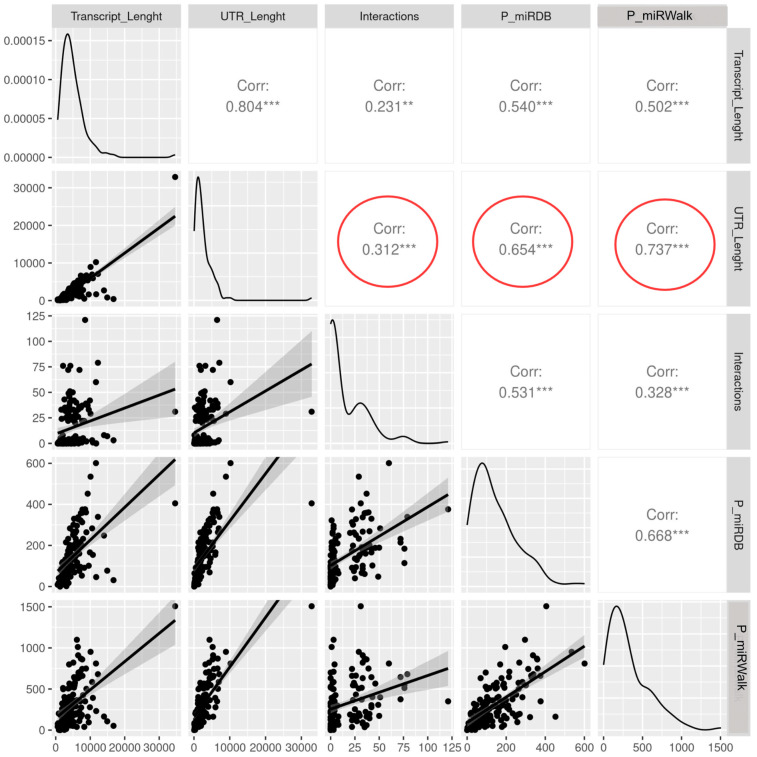
Correlation analysis showing the relationship between the UTR length of a transcript and the number of interacting microRNAs. The number indicated in the square is the value of the coefficient of correlation r, which ranges from −1 to +1. Two asterisks indicate *p* value < 0.01; three asterisks *p* value < 0.001. Red circles highlight the relationship between the UTR length and the number of verified interacting miRNAs (0.312), the number of miRNAs predicted with miRDB (0.654) or miRWalk (0.737).

As expected, the two predictors returned some common as well as some different microRNAs, and, in both cases, we found a significant positive correlation between the UTR lengths and the number of predicted binding sites (r = 0.654 for miRDB and 0.737 for MirWalk, Figure 7). On the contrary, when comparing the length of the UTR regions with the number of demonstrated interacting microRNAs, we found very low correlation (r = 0.312), suggesting that the true binding sites are selected and maintained, and do not depend on the length of the sequence, as a simple sequence consensus does. Surprisingly, several of the sequences predicted to have hundreds of potential interactors were demonstrated for any of them, generating an unexpected discrepancy between the number of predicted versus verified binders (Supplementary Table S5). The incompleteness of the interaction web, the presence of secondary structures and RNA binding proteins enhancing or inhibiting the binding may partially explain these differences.

## 4. Discussion

Since the relevance of the microRNA-mediated, post-transcriptional regulation is supported by evidence from thousands of studies, in normal cells and pathologies, a paradox must be addressed, as the amount of protein or mRNA that decreases in response to the expression of a microRNA is quite limited, and the rate of repression is usually in the range of 2–3 folds, making it difficult to explain the phenotypes observed in microRNA knockdowns [12,60]. This points to the possibility that microRNAs exert their most evident functions regulating key genes such as master transcription factors, and that they may act on multiple genes involved in the same process or pathway, thus potentiating the final effect. This is consistent with previous observations, obtained from a predicted microRNA-mRNA network, where extensive interactions between microRNAs and transcription factors were highlighted [45]. Notably, feed-forward loops were also shown, with transcription factors regulating microRNAs that downregulate them, or both co-regulating a third protein [61]. For example, Myc activates the expression of the transcription factor E2F1 and of the miR-17 cluster, composed of six miRNAs, comprising miR-17, miR18a, miR19a, miR20, miR19b and miR92 [62], which, in turn, control E2F1 level [63]. Genome-wide profiling of the transcriptome will help in elucidating which regulatory proteins control microRNA expression, better clarifying the extent of the cross-talk [64]

In this study, the analysis of a collection of trustworthy targets has confirmed that microRNAs finely control driver transcription factors and other key regulatory proteins. In fact, we show a remarkable enrichment of transcription factors among highly regulated mRNAs, yet limited to a defined subgroup of genes, possibly those determining the cell fate. For example, Zeb, Twist1 and Snai1 have been recognised as EMT drivers [65], and all of them are controlled by more than 20 miRNAs (Supplementary Table S3).

Regulatory circuitries are commonly disrupted in cancer cells and in other diseases, so they are likely to represent optimal therapeutic targets. Indeed, many cancer-associated genetic mutations affect genes and DNA regions involved in the transcriptional and post-transcriptional control, such as transcription factors, signalling proteins, chromatin modifying enzymes or ncRNAs. Inhibitors of oncogenic transcription factors and oncomiRs have recently entered in clinical trials [13] so that elucidating the interplay between different levels of regulation is crucial to predict the effect of interfering drugs and molecules. The presence of multiple overlapping targets for microRNAs that are candidates for therapies (Figure 5, Supplementary Table S1) suggests the urgency of an in-depth understanding of the full picture of relationships.

The goal of completing a trustworthy microRNA-mRNA interaction network is still far from being achieved as the intersection between low-scale luciferase assay network (this study) and the network obtained with a high throughput CLASH approach [18] corresponds to less than 1% of the interactions, pointing to incompleteness on both sides, similarly to the first attempts to decipher PPI networks [66]. It should be noted that low-throughput experimental approaches always consider predicted interactions as a starting point for validation, thus introducing a bias that CLASH methods do not have. Notably high-throughput analyses reveal unexpected non-canonical binding that ought to be validated in the future.

On the other hand, information coming from high-throughput experiments and predictions weakens the reliability of result interpretation [20]. A similar problem, concerning the reliability of protein–protein interactions (PPI), was addressed in the last 20 years by the IMEx consortium [27], by defining guidelines for what constitutes experimental proof of binding and by adding a score of reliability to each interaction [67] which considers, among other factors, how many times it has been described in the literature. In the IntAct database, the same score was applied to evaluate microRNA interactions, but the amount of data concerning microRNAs is still too limited to allow for filtering [15]. Several PPI databases follow common principles to offer highly reliable data to the user [27]. In contrast, ncRNAs are mainly collected in independent resources, following different criteria not always clear to the user [68]. Moreover, the integration of gene regulatory networks (GRNs) with post-transcriptional regulation is still in its infancy [69]. As the relevance of this intricate and dynamic interplay, which determines cell fate, is clearly emerging, an in-depth understanding of the global and local architecture of the regulatory system is certainly needed.

## 5. Conclusions

Countless studies have contributed to elucidate the effects of microRNAs on specific genes, but very little is still certain about the extent of the regulation mediated by microRNAs and how it cooperates with the other layers of regulation. Transcriptional and post-transcriptional effects certainly contribute to the final expression level, but the knowledge in the two fields is still modestly interconnected [69]. Moreover, the excess of potential binding sites, largely exceeding the microRNA abundance, imply that the regulation may be sensitive to microRNA levels [9,70], which also should be taken into account when drawing models.

Despite the lack of knowledge on the biology of ncRNAs, from the regulation of their expression to their function, and even on their coherent classification, there is no doubt that these molecules have made a revolution in our understanding of the cell behaviour and must, from now on, be taken in consideration on any perturbation prediction.

## Data Availability

All data are available in public repositories.

## Supplementary Materials

The following supporting information can be downloaded at https://www.mdpi.com/article/10.3390/biology14010043/s1. Supplementary Figure S1: Diagram showing the relationships among GO terms enriched in genes regulated by more than 25 microRNAs. The diagram was drawn with the VLAD tool [38]. Supplementary Table S1: Complete list of nodes of the microRNA-mRNA network with the number of edges (degree value). The degree number was calculated by Cytoscape App [29]. The third sheet contains the full list of interactions considered. Supplementary Table S2: GO molecular function enrichment in hub genes in respect to the whole genome, according to BinGO, (*p* < 0.0005) [39]. Most significant terms, selected as representative for Figure 3 were highlighted in pale green or yellow. The two sheets contain the results for genes with 25 microRNAs (used in Figure 3) and with 20 microRNAs, respectively. The enrichment for the selected classes is confirmed in both. Supplementary Table S3: Comparison between the number of microRNAs regulating each transcription factor and the number of genes regulated by the transcription factor (retrieved from TFlink, “small scale dataset”). The master transcription factors are regulated by multiple microRNAs, while most of the others are not. Supplementary Table S4: Reactome pathways over-represented in 13 set of microRNA targets. The selected microRNAs all bind to more than 100 targets and have been described in the literature as OncomiR or a tumour suppressor (see Table 1). A pathway analysis tool was used [40] and the 20 most significant pathways according to the FDR score were reported. Supplementary Table S5: The 3′ UTR lengths of a sample of transcripts, compared with the number of predicted and verified interactors.
