# Supplementary figures and images for: Integrated Analysis of microRNA Targets Reveals New Insights into Transcriptional–Post-Transcriptional Regulatory Cross-Talk
Source: Biology (Basel). 2025 Jan 8;14(1):43. doi: 10.3390/biology14010043 (PMC11762646; doi:10.3390/biology14010043)

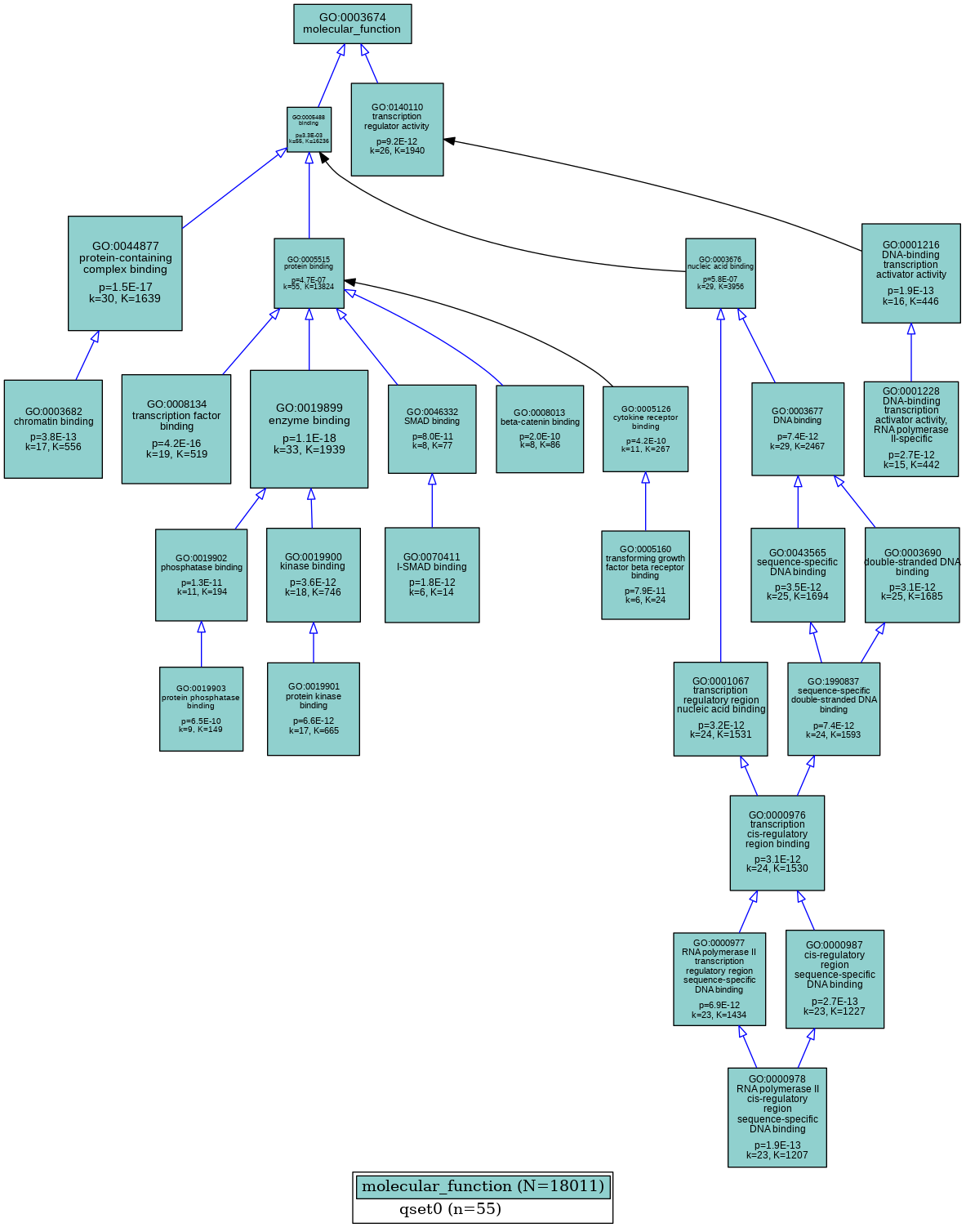

Supplement: Supplementary file 1 [file biology-14-00043-s001.zip › Supplementary figure 1.png]
